# Characterizing human genomic coevolution in locus-gene regulatory interactions

**DOI:** 10.1186/s13040-019-0195-y

**Published:** 2019-03-15

**Authors:** Daniel Savel, Mehmet Koyutürk

**Affiliations:** 1 Department of Electrical Engineering and Computer Science, Case Western Reserve University, 10900 Euclid Avenue, Cleveland, 44106 OH USA; 2 Center for Proteomics and Bioinformatics, Case Western Reserve University, 10900 Euclid Avenue, Cleveland, 44106 OH USA

**Keywords:** Coevolution, Co-conservation, Phylogenetic profile, Multiple hypothesis testing, eQTL

## Abstract

**Background:**

Coevolution has been used to identify and predict interactions and functional relationships between proteins of many different organisms including humans. Current efforts in annotating the human genome increasingly show that non-coding DNA sequence has important functional and regulatory interactions. Furthermore, regulatory elements do not necessarily reside in close proximity of the coding region for their target genes.

**Results:**

We characterize coevolution as it appears in locus-gene interactions in the human genome, focusing on expression Quantitative Trait - Locus (eQTL) interactions. Our results show that in these interactions the conservation status of the loci is predictive of the conservation status of their target genes. Furthermore, comparing the phylogenetic histories of intra-chromosomal pairs of loci and transcription start sites, we find that pairs that appear coevolved are enriched for cis-eQTL interactions. Exploring this property we found that coevolution might be useful in prioritizing association tests in cis-eQTL detection.

**Conclusions:**

The relationship between the conservation status of pairs of loci and protein coding transcription start sites reveal correlations with regulatory interactions. Pairs that appear coevolved are enriched for intra-chromosomal regulatory interactions, thus our results suggest that measures of coevolution can be useful for prediction and detection of new interactions. Measures of coevolution are genome-wide and could potentially be used to prioritize the detection of distant or inter-chromosomal interactions such as trans-eQTL interactions in the human genome.

**Electronic supplementary material:**

The online version of this article (10.1186/s13040-019-0195-y) contains supplementary material, which is available to authorized users.

## Introduction

Advances in DNA sequencing have enabled the assembly of high quality and complete genomes of many different organisms, as well as the reassembly and refinement of whole genomes. As of particular interest, the human genome has been through many iterations improving its quality and completeness. Even though the entire sequence representing the population is known, the identification of functional units and regulatory elements, and the interactions between these elements is largely incomplete.

Comparative genomics is used to predict functional units, and it is one of the primary whole genome scale methods. High rates of conservation have been shown to be indicative of functionality [1], and several tools have been developed to assess evolutionary conservation and use this information to identify functional elements [2, 3]. Initiatives such as the ENCODE project [4] have made large strides into identifying the functional units of the human genome. However, the information uncovered by projects like ENCODE is largely limited to the identities and location of these functional elements and do not capture the relationship between these elements and their target genes.

In the literature, pairwise rates of conservation have been used commonly to identify protein-protein interactions (PPIs) and characterize protein function [5]. Protein coevolution refers to the observed correlation in the conservation patterns of two or more proteins across a wide range of organisms, and has been repeatedly shown to provide valuable information on the interactions and functional association among those proteins [6, 7]. Many methods have been developed to accurately characterize the coevolution of two or more proteins and to effectively use this information to predict interactions among proteins [8, 9].

In this paper, we stipulate that the functional interactions that can be captured by coevolution may include locus-gene regulatory interactions. It has been increasingly shown that non-coding DNA sequence has function, and like protein-coding sequences, highly conserved non-coding sequences are likely to have functionality [10]. Motivated by this observation, we characterize coevolution in the context of locus-gene interactions with a focus on expression quantitative trait loci (eQTL) interactions. An eQTL interaction is defined as the statistical association between a genomic locus, usually the allele status of a single nucleotide polymorphism (SNP), and the level of expression of a gene. eQTL interactions are considered indicators of potential regulatory interactions between the corresponding genomic locus and gene. In the next subsections, we discuss the literature on the use of protein coevolution in the characterization of protein function and elaborate on eQTL interactions.

### Protein coevolution

Coevolution refers to the observation that the evolutionary conservation of two or more functionally associated cellular elements is correlated across a range of species. A plausible explanation to such correlation is that there is a functional relationship driving the selective pressure to conserve the pair of units together [11]. Coevolution has been well-defined and well-studied in the context of the evolution of proteins and their domains. For example, contact domains of proteins forming a complex need to be complementary in order for the complex to form, so variations in only one of the domains could inhibit that interaction. Furthermore, proteins commonly participate in pathways where the proteins work either in conjunction or succession to complete some higher level function, where the absence of one of the proteins could lead to loss of function.

In order to assess protein coevolution, the evolutionary histories of the proteins need to be constructed. A common and simple way of representing evolutionary histories is to use phylogenetic profiles, which represent the level of conservation of the protein in a set of organisms. Typically, phylogenetic profiles comprise alignment scores between a protein of interest and its homologs. These alignment scores are obtained using either already known sets of protein families or a search tool like the protein specific version of BLAST [12], by comparing each protein’s sequence against a library of “reference" genomes. Once the profiles are constructed, the coevolution of all pairs of proteins is assessed based on the correlation between these phylogenetic profiles. Information theoretic measures are commonly used to asses the correlations (or statistical dependency) among phylogenetic profiles.

An important distinction between different methods for coevolution detection is based on the resolution at which the profiles are generated and or comparisons are performed. The resolutions break down to three general levels: 1) whole protein [5], 2) domain [13], and 3) residue level [14]. Earlier methods work at the resolution of whole proteins; the presence or absence of a sufficiently similar matching protein in each of the reference genomes would act as the phylogenetic profile for that protein. These profiles are visualized and stored as simple discrete-valued vectors, so the coevolution search consists of finding similar pairs of vectors. Since domains may evolve independently from the rest of the protein, methods that utilize phylogenetic profiles at the resolution of domains improve upon these methods by capturing the evolution of coding sequences at a higher resolution [13, 15]. However, since the domains of many proteins are not characterized, some methods assess coevolution at the level of residues, thereby enabling the identification of domains based on their conservation [14, 16]. Using alignment scores or transformed BLAST E-values can also be used in place of a simple boolean for hit or miss in a genome [17].

Once the phylogenetic profiles are generated, the process of identifying coevolution becomes the process of identifying similar patterns. One common way of defining similarity for this process is that a pair of profiles with high mutual information [18] is likely to be coevolved. When assessing coevolution at residue level resolution, it is possible to capture intra-protein coevolution as well. It has been shown that residues that are in contact with eachother in the folded structure of a protein could be predicted by examining the mutual information of those bases within a multiple sequence alignment [19].

In order to better represent a protein-specific phylogeny, phylogenetic tree based methods were also developed [16, 20]. For example, a family of methods referred to as *mirrortree* use multiple sequence alignment to build a phylogenetic tree for each protein. These methods then measure the correlation of the phylogenetic trees rather than vectors to quantify and detect coevolution. In practice, rather than actually constructing the phylogenetic tree for the protein, the methods generate a distance matrix that stores all pairwise alignments between protein sequences that are incident on the multiple sequence alignment. In the more recent methods, tree generation software is applied to the multiple sequence alignment and distance matrices are extracted from these generated trees. These distance matrices that are generated could be used by any tree generation method to construct a tree, but *mirrortree* methods instead directly compare the distance matrices of pairs of proteins rather than pairs of trees that would have been generated from those matrices. MATRIXMATCHMAKER is another method that was developed to detect coevolution between pairs of proteins that also utilizes distance matrices, but is more computationally intensive [9]. The MATRIXMATCHMAKER method was improved upon to help address the increased computational complexity [21].

The principle of coevolution has been further studied beyond interactions between pairs of proteins or genes. It has been shown that physical interactions between proteins and DNA sequence exhibit coevolution, such as those between transcription factors and their binding sites [22]. There have also been explorations into the coevolution patterns between proteins and non-coding RNA sequences, such as miRNA [23]. These studies have explored the coevolution patterns present in these interactions, but did not use coevolution metrics in a predictive manner.

Recently, direct coupling analysis (DCA) based approaches have been used to predict protein residue contact sites [24, 25] and epistatic pairs of SNPs in bacterial genomes [26, 27]. These DCA methods utilize the principle of maximum entropy as the metric to score pairs of loci.

An important consideration for coevolutionary analyses is the selection of organisms from which to generate the phylogenetic profiles or distance matrices. Typically, as the number of genomes used in the analysis increases, predictive power also increases [28]. Besides the number of genomes that are used in the analysis, the evolutionary relationships among the genomes used can also have an effect on the predictive performance [29].

### eQTL interactions

An eQTL interaction between a genomic locus and a gene represents the statistical association between the genotype of the SNP at that locus and the mRNA-level expression of the gene. There are two sub-classes of eQTL interactions, cis-eQTL and trans-eQTL interactions. The definition of these sub-classes relies entirely on the genetic distance between the SNP and the target gene. A cis-eQTL interaction is between a SNP and a gene that is on the same chromosome and is nearby. On the other hand, a trans-eQTL is between a SNP and either a gene on the same chromosome that is distant or a gene that is on a different chromosome. For example, the GTEx project, which is the primary repository of detected eQTL interactions in the human genome, considers SNPs that are within 1 Mbp of the target gene to be a cis-eQTL [30].

Two contemporary tools that are used to perform large numbers of association tests are Matrix eQTL [31] and FastQTL [32]. FastQTL is a more recent tool that was designed to only test potential cis-eQTLs, and Matrix eQTL can test for either cis- or trans-eQTLs. One issue that arises with eQTL detection is the large number of association tests that are performed. Particularly, during trans-eQTL detection the number of tests can exceed 10^12^, comparing nearly all SNPs against all genes. This large number of performed association tests necessitates the use of multiple hypothesis testing (MHT) correction. One of the methods used for MHT correction is the Benjamini-Hochberg procedure which is a Bonferroni-like correction process which is not as conservative as a Bonferroni correction. The method can be implemented in an iterative manner. Initially a strict *p*-value threshold of significance is calculated from a specified false discovery rate and the number of tests performed. Hypotheses with nominal *p*-values that exceed this threshold are rejected, and the significance threshold is relaxed slightly for each of these rejected hypotheses. In turn, more tests can pass this relaxed threshold. The process of rejecting hypotheses and adjusting the threshold continues until no more tests exceed the adjusted threshold.

In this work we explore the evolutionary relationship between SNPs and genes involved in cis-eQTL interactions. We examine the co-conservation patterns between them, and we use a *mirrortree*-based method to characterize the coevolution patterns of those same pairs. Further we show that we can leverage these coevolution patterns to aid in the prioritization of eQTL detection.

## Results and discussion

In this section we present our results which are broken down into three subsections. The first subsection focuses on the relationship between the conservation status of SNP and gene pairs that form eQTL interactions. The second subsection characterizes the coevolutionary relationship of the interacting SNP-gene pairs, as assessed using their phylogenetic histories. Then we explore using this coevolutionary relationship to prioritize eQTL detection in the third subsection.

### Conservation of SNPs and genes involved in eQTL interactions

**Fig. 1:**
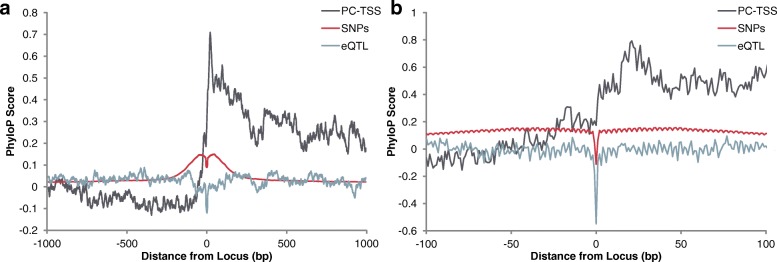
Patterns of conservation. **a** Average rates of conservation of the 1000 bases upstream and downstream of protein-coding transcription start sites (PC-TSSs), SNP loci, and SNP loci involved in at least one eQTL interaction. **b** Average rates of conservation of the 100 bases upstream and downstream of the same sets of loci

We profiled the genomic conservation of the flanking sequence for SNP loci and transcription start sites that take part in eQTL interactions. Below, in the “Materials and methods” section we describe in detail how these profiles are constructed. We also constructed profiles for all SNPs in the human genome that are reported in version 146 of the dbSNP database [33]. Figure 1 displays a normalized pileup of the PhyloP [34] scores of the bases surrounding SNP loci associated with eQTL interactions, all SNP loci, and the transcription start sites of eQTL target genes. The PhyloP scores represent deviation from a neutral rate of evolution. Bases with positive scores are bases that exhibit conservation, a lower than expected rate of mutation. Bases with negative scores are those that exhibit accelerated evolution, a higher than expected rate of mutation. The magnitude of the scores is proportional to the difference between expected and exhibited rate of mutation.

We found that there is typically a conserved region surrounding the locus that harbors the SNP and that the locus that harbors the SNP is undergoing accelerated evolution (negative PhyloP conservation score). SNPs are less conserved than transcription start sites (*p*< 2.534×10^−9^) and furthermore SNPs involved in eQTLs are less conserved than SNPs in general (*p*< 2.2×10^−16^). Both of these *p*-values were calculated using the one-sided Kolmogorov-Smirnov (KS) test.

**Fig. 2:**
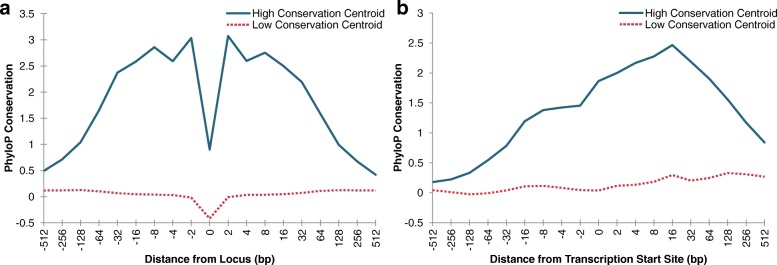
Clustering centroids. Centroids of clusters identified by clustering conservation profiles of (**a**) SNPs and (**b**) transcription start sites of genes involved in at least one eQTL interaction. Clustering was performed with K-means clustering, using K = 2

In order to identify patterns of conservation exhibited by SNPs and genes that are involved in eQTL interactions, we clustered the conservation profiles of SNPs and genes using the K-means algorithm. We created vectors that contain the PhyloP scores of conservation of the upstream and downstream bases of the loci to serve as the conservation profiles. These profiles are described in further detail in the “Materials and methods” section. From this clustering analysis, we found that for both SNPs and genes the best clustering occured when using only two centroids, as determined by considering cluster membership sizes, sums of distances, and recurring centroid patterns (Figure S2 and S3 in the Additional file 1). Figure 2a shows the centroids of the two clusters identified by clustering the SNP profiles. We found that one centroid tended to stay around PhyloP scores of 0, indicating little to no conservation and little to no acceleration compared against a neutral rate of evolution. The other dominant centroid pattern that consistently appeared during clustering, was a centroid that exhibited conservation in the flanking sequence of the SNP locus. Based on these distinct conservation patterns, we refer to these centroids respectively as the low conservation centroid and the high conservation centroid. Both centroids suggest that the SNP locus has a lower rate of conservation than its immediate flanking sequence.

Clustering the gene profiles also produced two clusters: one with a centroid exhibiting a neutral rate of evolution and one with a centroid that displays higher rates of conservation. Figure 2b shows these two centroids. However, in contrast to the high conservation SNP centroid, the high conservation gene centroid does not exhibit a symmetric pattern of conservation surrounding the transcription start site, it rather shows increasing conservation leading up to and past the transcription site. As expected, exon sequence of the genes exhibit higher rates of conservation than upstream sequence.

To examine the evolutionary interplay between the SNPs and their target genes in eQTL interactions, we compared their levels of conservation as classified based on the patterns identified using cluster analysis. Table 1 is a contingency table of eQTL interactions based on the cluster assignment of the SNP and the cluster assignment of the target gene that is involved in that interaction. We performed a chi-squared test on this contingency table to test whether highly conserved SNPs are more likely to have eQTL interactions with highly conserved genes. We found that *χ*^2^=23.651(*p*< 1.155×10^−6^). This result suggests that for eQTL interactions the conservation status of a SNP is correlated with the conservation status of its target gene, with high conservation SNPs interacting with high conservation genes.

**Table 1:** Conservation contingency table

	HC SNP	LC SNP	Marginal totals
HC target gene	428	7544	7972
LC target gene	1775	40975	42750
Marginal totals	2203	48519	50722

HC and LC refer to high conservation and low conservation respectively, and SNPs and genes are assigned to these groups based on their cluster identity as determined by the clustering analysis

### Coevolution of SNPs and genes involved in eQTL interactions

To further characterize the evolutionary relationship between SNPs and their target genes in eQTL interactions, we also examined and compared their phylogenetic histories. For this purpose, we used a *mirrortree* based method to calculate a measure of coevolution for each SNP-gene pair. While DCA based methods have been used recently to quantify coevolution in locus-locus interactions, they have been used on relatively small sequences compared to that of the human genome. The primary data source for phylogenetic information is a library whole genome multiple sequence alignment of 100 vertebrate genomes provided publicly by the UCSC Genome Browser [35]. Subsequences of the multiple sequence alignment were used to assess the phylogenetic history of the SNPs and genes that participate in eQTL interactions. Another reason we use *mirrortree* over DCA is that we are quantifying the coevolution between between pairs of sequences rather than pairs of loci alone. This process is described in detail in the “Materials and methods” section.

**Fig. 3:**
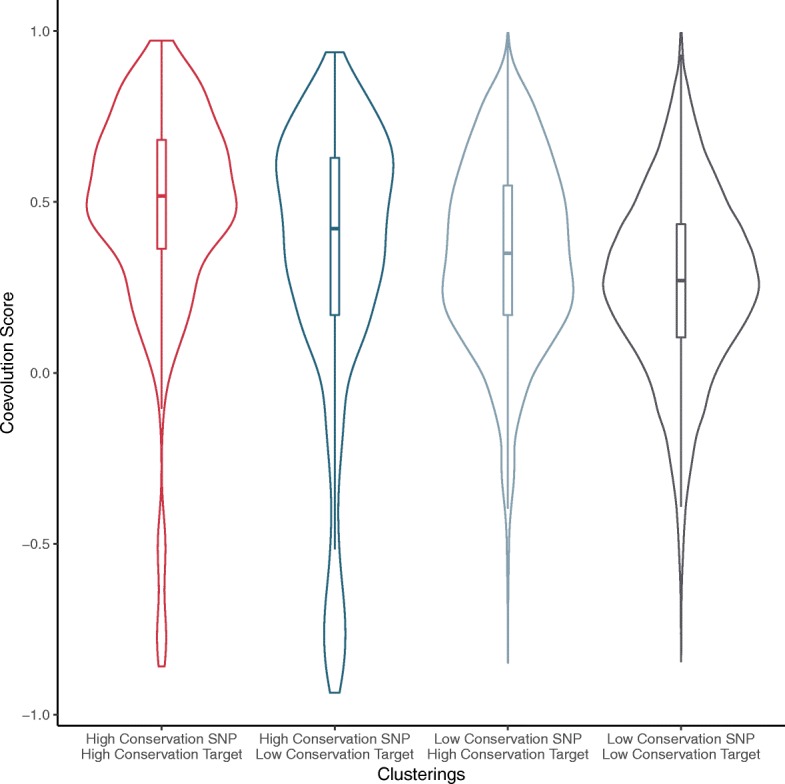
Coevolution of co-conservation classes. Distributions of coevolution scores of the different sets of eQTL interactions as classified by the conservation clustering analysis

To examine the relationship between our conservation metrics described above and the coevolution metrics we describe here, we calculated the coevolution scores for all eQTL pairs that we classifed using the clustering approach. Figure 3 shows the distributions of coevolution scores for each class of eQTL interaction. We found that the interactions with high conservation SNPs interacting with high conservation genes, had the highest levels of coevolution score, while poorly conserved SNPs interacting with poorly conserved genes had the lowest relative coevolution scores. Table 2 provides the *p*-values for all pairwise comparisons of these distributions.

**Table 2:** Co-conservation class comparison table. *P*-values for comparisons of the distributions of coevolution scores for each possible pairing of co-conservation classes of eQTL interactions (calculated using the two-sided KS-test)

Class 1	Class 2	*p*-value
High conservation SNP	High conservation SNP	
High conservation target	Low conservation target	*p*<1.045×10^−6^
High conservation SNP	Low conservation SNP	
High conservation target	High conservation target	*p*<1.11×10^−15^
High conservation SNP	Low conservation SNP	
High conservation target	Low conservation target	*p*<2.2×10^−16^
High conservation SNP	Low conservation SNP	
Low conservation target	High conservation target	*p*<8.946×10^−9^
High conservation SNP	Low conservation SNP	
Low conservation target	Low conservation target	*p*<2.2×10^−16^
Low conservation SNP	Low conservation SNP	
High conservation target	Low conservation target	*p*<2.2×10^−16^

**Fig. 4:**
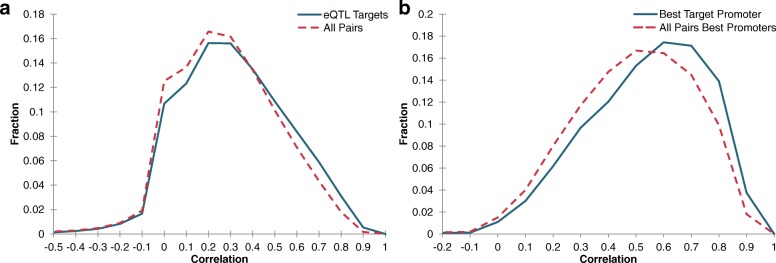
Coevolution score distributions. **a** Distribution of coevolution scores of SNP-TSS pairs involved in eQTL interactions compared against a background distribution. **b** Coevolution score distribution of eQTL interactions when pairing SNPs with promoters compared against a background distribution

We quantified the coevolution of the SNPs and genes involved in eQTL interactions, and compared the distribution of these scores against a background distribution. The background distribution was generated by first identifying all SNPs and all genes that take part in at least one eQTL interaction. We scored all possible pairs of these identified SNPs and genes, namely about 511 million SNP-gene pairs, to form the background distribution. Figure 4a shows a comparison of the normalized histograms of eQTL interactions against the background. We found that eQTL interactions exhibit higher levels of coevolution than arbitrary pairs of SNPs and genes (*p*< 1×10^−300^, two-sided KS-test). The higher levels of correlated phylogenetic history suggests that eQTL interactions may impart evolutionary pressure towards co-conservation.

Motivated by this result, we also explored the coevolutionary relationship of eQTLs with regard to known regulatory elements of the target genes. For this analysis, rather than comparing the SNP phylogenetic history with that of the gene, we compared them against the phylogenetic history of promoters associated with the target gene. The promoters are those that were discovered as part of the ENCODE project, and we acquired them through the Ensembl project [36]. A challenge here is that often there are multiple promoters that map to the same gene, so there is a one-to-many mapping when identifying promoters for a single gene. To provide a well-defined measure of coevolution between a gene’s promoters and a SNP, we hypothesized that the eQTL interaction occurs through a single promoter of its target gene. Based on this hypothesis, for each eQTL interaction, we computed the coevolution score between the SNP and each of the gene’s promoters. Subsequently, we reported the score of the promoter with highest coevolution score as the coevolution score of the SNP and the gene’s promoters. Again, we compared the distribution of these coevolution scores against a background distribution. In order to accurately assess the significance of this distribution, we constructed a background distribution from the coevolution scores between each SNP and the promoters of each gene, by taking the maximum coevolution score between the SNP and each of the promoters. In this case, the number of pairs that form the background distribution are consistent with those used to generate the background distribution for SNP-gene pairs. Figure 4b, shows the comparison between the normalized histograms of eQTL interactions and the background. Again, SNP-promoter pairs that are associated with eQTL interactions exhibit higher levels of coevolution score as compared to the background distribution (*p*< 1×10^−300^, two-sided KS-test). Notably, the SNP-promoter background distribution is shifted towards higher rates of correlation than the SNP-gene background distribution, which is consistent with how each recorded score was chosen as the highest correlation of a set of pairs.

We examined the relationship between minor allele frequency and coevolution score, to see if there was a correlation between high variation within the human population and coevolution scores. We found that there was no discernable difference between the distributions of minor allele frequencies for different ranges of coevolution scores.

### Using coevolution to prioritize eQTL detection

As shown above, eQTL interactions are more likely to exhibit correlated phylogenetic histories as compared to random pairs of sequences. Motivated by this observation, in order to explore the utility of genomic coevolution in detecting regulatory interactions, we applied coevolution to the prioritization of eQTL interactions. We used data made available by the GTEX project [30], V6, as they provide the nominal *p*-values of all cis-eQTL tests performed and not just those that are deemed significant. Raw data for the analysis as well as genotype data was provided via dbGaP, phs000424.v6.p1. With this data we are able to apply the multiple hypothesis testing (MHT) correction process on the original dataset. For each cis-eQTL SNP-Gene pair tested in the GTEX project, we calculated the genomic coevolution between the SNP and the target gene. We prioritized eQTL detection by focusing on SNP-Gene pairs that exhibit high levels of coevolution. By thresholding on the coevolution score we identified sets of the SNP-Gene pairs for different levels of coevolution. For each of these sets, we applied the MHT correction process to them and reported the *p*-value threshold of significance. A property of this MHT correction process is that the ratio of the significance threshold relaxed by prioritizing with coevolution score and the non-prioritized significance threshold is directly proportional to the level of enrichment that SNP-gene pairs with that coevolution score have for significant eQTL interactions. We expand on this property in the “Materials and methods” section.

**Fig. 5:**
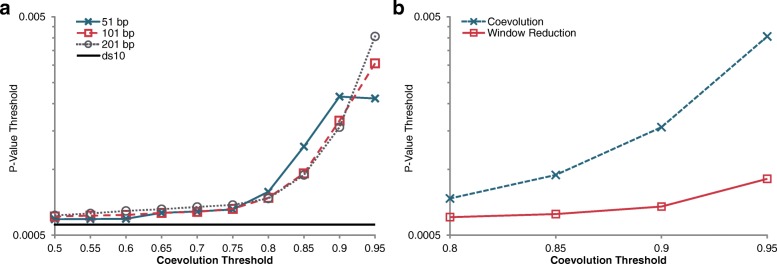
eQTL detection prioritization. **a** Prioritizing eQTL detection with coevolution scores relaxes the significance threshold. Results are shown for using different size sequences to calculate coevolution score. The bottom line represents randomly downsampling the data to 10% remaining rather than prioritizing with coevolution score. **b** The relaxation of the significance threshold can only be partially explained by the reduction of the average distance of tested pairs

Figure 5a shows how prioritizing the eQTL tests by coevolution affects the *p*-value significance threshold. We also tested whether random downsampling to reduce the number of tests performed affected the *p*-value threshold of significance and confirmed that it does not affect the significance threshold due to how the Benjamini-Hochberg method of MHT correction works. The significance threshold was 5.61×10^−4^ for the full dataset and when reducing the number of tests by a factor of 10 using the random downsampling, the significance threshold was nearly unchanged at 5.59×10^−4^. We found that when prioritizing the eQTL tests with coevolution the significance threshold is relaxed, and we found that the stricter the coevolution thresholds that are used the more relaxed the significance threshold becomes. This effect became apparent for SNP-gene pairs with coevolution scores > 0.75, indicating strongly correlated phylogenetic histories. At the highest level of coevolution threshold, the significance threshold was relaxed to 2.11×10^−3^. This shows that SNP-gene pairs that have very high levels of correlated phylogenetic histories are enriched for eQTL interactions by over 7 times, SNP-gene pairs with coevolution score of 0.95 or greater are more than 7 times as likely to be a significant eQTL interaction as compared to an arbitrary SNP-gene pair.

A side effect of prioritizing eQTL tests by genomic coevolution is that due to a correlation between genomic distance and observed rate of coevolution there is a reduction in the average distance of the tested pairs of SNPs and genes. Prioritizing eQTL tests by genomic distance is effective, as it has been shown that the regions closer to the transcription start sites are enriched for SNPs that participate in eQTL interactions [37]. Taking this observation into account, we explored how much of the relaxation in the significance threshold is due to this side effect as opposed to evolutionary pressures. For different coevolution score thresholds, we calculated the average distance of the tested SNP-gene pairs, and performed a distance based prioritization such that the average distance of the tested pairs was the same as prioritizing for a given coevolution score threshold. Figure 5b shows the comparison of these two prioritization methods. We found that even though reducing the average distance of tested SNP-gene pairs relaxed the significance threshold the threshold was relaxed to a greater degree when using a comparable coevolution score based prioritization. So we found that there was some interplay between coevolution score prioritization reducing the average distance of tested pairs and this reduced average distance contributing to the relaxation of the significance threshold. However, the relaxation of significance threshold cannot be entirely explained by this reduction in test distance, and the interplay is quite minimal until very high levels of coevolution threshold are used, coevolution scores ≥ 0.95.

## Conclusion

We characterized the co-conservation and coevolution of locus-gene regulatory interactions in the human genome. We discovered with high confidence that there is interplay between the conservation status of SNPs and their target genes in eQTL interactions. Furthermore, using subsequences of a whole genome multiple sequence alignment we were able to asses the correlation between the phylogenetic histories of those same eQTL interactions, and we discovered that pairs of SNPs and genes that appear coevolved are enriched for eQTL interactions. We applied this property to the prioritization of eQTL association tests and found that lower signficance eQTL interactions could be identified.

Coevolution can be used to create genome-wide metrics, and could potentially provide utility in identifying distant and inter-chromosomal interactions such as trans-eQTL interactions in the human genome. Further work in this area includes assessing trans-eQTL coevolution as we did for cis-eQTLs, and examining the interplay between coevolution and the robustness of eQTL detection with regards to sample set size. Furthermore, it may be beneficial to explore other metrics of coevolution such as mutual information or direct coupling analysis in the context of locus-gene interactions in the human genome.

## Materials and methods

We compare the genomes of a large set of organisms to quantify the levels of conservation of human DNA sequence and the levels of coevolution between pairs of sequences. In order to accurately map the evolutionary history of each DNA segment we use a whole genome multiple sequence alignment. This process is computationally intensive, so we use a publically available dataset provided by the UCSC Genome Browser [35]. The dataset is a pre-calculated whole genome multiple sequence alignment of 100 vertebrates. The types of organisms range from primates and mammals to birds and fish; UCSC genome browser also provides pre-calculated base-by-base conservation scores using two different methods, PhyloP [34] and PhastCons [38]. Another available source of base-by-base conservation scores is GERP++ [39]. We use both the whole genome multiple sequence alignment and the base conservation score data are for both a co-conservation analysis and for a coevolution analysis.

### Genomic conservation profiles

We first performed a co-conservation analysis of the eQTL interaction data in a manner similar to an analysis done for miRNA binding [23]. For this analysis we created profiles for the associated SNPs and target genes using the pre-calculated PhyloP data. We use PhyloP scores for our base conservation scores as it treats each base independently, and since we are interested in specific loci such as SNPs we want the base score to represent that locus alone. Both PhastCons and GERP++ use a Markov process to calculate the conservation score for each base, so base scores are influenced by nearby bases. Furthermore, PhastCons and GERP++ both quantify only conservation, while PhyloP quantifies both conservation and acceleration which we see present at known sites of common genomic variation. Each profile consists of the base conservation scores of the SNP locus, for the eQTL profile, or of the transcription start site, for genes, as well as the conservation scores of the 1000 base pairs upstream and downstream. This generates profiles that consist of 2001 base conservation scores. In order to reduce the effects of high dimensionality on our analysis we perform dimensionality reduction by binning and averaging scores together in a logarithmic manner. The bases that are closest to the loci of interest are in smaller bins than those that are further way, giving more weight to the resulting reduced dimension profile. For example, the conservation score of the locus of interest is in a bin by itself, the scores for the two bases upstream of the locus are binned together, and the four scores upstream from those are binned together. By binning the conservation scores in this manner we reduce the dimensionality of the profiles from 2001 down to 19. We performed our clustering analysis on these 19-dimension profiles using k-means clustering.

### Creating Phylogenetic matrices

As we discussed above, state-of-art methods for analyzing coevolution make use of distance matrices that are generated from multiple sequence alignments of homologous proteins. We applied this principle to genomic sequence using multiple sequence alignments of homologous DNA sequence rather than protein sequence. One method of acquiring the homologous DNA sequences is to use BLAST against a library of genomes, but this process can produce a number of false positives. This can be exacerbated when the library of genomes is large and when the query sequences are small. To remove the effect of querying relatively small sequences we use whole genome multiple sequence alignments to identify homologous sequences. In this manner the number of homologous sequences for a DNA segment is stable when querying different size segments. In order to create a distance matrix for a genomic segment, we extract a subsequence from the whole genome multiple sequence alignment and further extract all the pairwise alignments in order to calculate the distance matrix. The process of extracting the subsequences of the whole genome multiple sequence alignment is performed serially, and requires just a single pass through the alignment file. And each distance matrix is generated by comparing all pairs of sequences that are contained in that subsequence of the multiple sequence alignment which can subsequences from as many as 100 genomes. In our generated matrices, we store the alignment scores of all pairwise alignments that are induced by the multiple sequence alignment, so it is more accurate to state that we are generating similarity matrices as high nominal values indicate high sequence match rates. We evaluated coevolution with these matrices using a *mirrortree* based method.

### BipartiteMirrorTree and sparse correlation

In order to evaluate the coevolution of pairs of sequences that belong to two distinct classes we developed a modification to *mirrortree*, that we refer to as BIPARTITEMIRRORTREE. BIPARTITEMIRRORTREE uses the sparse correlation of similarity matrices in order to measure the rate of coevolution between the pairs of sequences those matrices described; BIPARTITEMIRRORTREE can characterize the coevolution between all pairs that can be formed from two distinct sets, such as SNPs and genes. Since we characterized eQTL interactions, which are associations between SNPs and genes, we used BIPARTITEMIRRORTREE to characterize the coevolution of all previously identified eQTL interactions as well as all possible pairs of SNPs and genes. The general runtime complexity of BIPARTITEMIRRORTREE is O(*sgl*^2^), where *s* is the number of SNPs, *g* is the number of genes, and *l* is the number of genomes in the multiple sequence alignment used to create the similarity matrices. However, for cis-eQTL detection all SNPs are not paired with all genes; genes are only paired with SNPs that are within 1mbp. In this instance the number of SNP-gene pairs is much less than all possible pairs, *sg*, but it is still much greater than the size of the similarity matrices, *l*^2^. For these cis-eQTL analyses, the practical runtime complexity of BIPARTITEMIRRORTREE is O(*n*), with *n* being the number of SNP-gene pairs examined.

As we stated above we use sparse correlation as our measure of coevolution. Previous implementations of *mirrortree* use full correlation as the measure of coevolution. One characteristic of using full correlation, is that when there are large amounts of missing alignments (due to either poorly conserved sequence or poor mapping) in both matrices, the correlation of the two matrices will be inflated. For example, as we are using similarity matrices a missing alignment or a poor alignment would lead to a pairwise similarity score of 0, and a large number of them in a pair of matrices can lead to many spurious matches between the two matrices. These spurious matches will in turn inflate the correlation between the pair of matrices, and they will appear to be coevolved when they might not be. Therefore, BIPARTITEMIRRORTREE uses sparse correlation which better captures the idea of coevolution and does not become inflated with missing data. One of the reasons we used similarity matrices as opposed to distance matrices, is that they lead to an elegant definition and implemenation of sparse correlation: while calculating the correlation between a pair of similarity matrices ignore instances when the corresponding values in both matrices are 0.

### Relationship between significance threshold and enrichment

The way the Benjamini-Hochberg procedure performs multiple hypothesis testing correction gives insight into the enrichment exhibited by prioritization. The unprioritized procedure works as follows:

1. Let *α* be the desired false discovery rate (usually 0.05)
2. Let *H*_0_...*H*_*n*_ be the set of hypotheses (e.x. locus-gene pairs to be tested for eQTL interactions)
3. Let *P*_0_...*P*_*n*_ be the set of corresponding nominal *p*-values (e.x. the *p*-value of association between the genotype of the locus and the expression of the gene)
4. Sort the hypotheses (e.x. locus-gene pairs) in ascending order of *p*-values
5. Find the largest K such that $P_{K} < \frac {K\alpha }{n}$
6. Reject hypotheses *H*_0_...*H*_*K*_ (e.x. identify locus-gene pairs *H*_0_...*H*_*K*_ as significant eQTL interactions)

In this case, the *p*-value significance threshold becomes, P = $\frac {K\alpha }{n}$, for the full set of hypotheses. However, prioritizing with a threshold (e.x. coevolution score) effects the set of hypotheses. For a given threshold, let *β* be the fraction of all significant interactions that exceed that threshold (e.x. eQTL interactions with high coevolution scores), and let *γ* be the fraction of all hypotheses that exceed that threshold (e.x. locus-gene pairs with high coevolution scores). During prioritization we can then express the *p*-value significance threshold as a modified version of the significance threshold for non-prioritized hypotheses, $P'=\frac {\beta K \alpha }{\gamma n}$. With this formulation, as long as *β*>*γ* then *P*^′^>*P*, thus the significance threshold is relaxed. Furthermore, in this case $\frac {P^{\prime }}{P} = \frac {\beta }{\gamma }$, thus the ratio of *P*^′^ and *P* provides a measure of the enrichment a given prioritization threshold has.

## Additional file

Additional file 1 **Figure S1**: First Singular Vector of SVD analysis compared to the High Conservation Centroid of the clustering analysis. **Figure S2**: Centroids when using different values of K during clustering. **Figure S3**: Within-Cluster Distance for different values of K during clustering. **Figure S4**: Distributions of the distance between pairs of SNPs and target gene promoters. (PDF 120 kb)

## References

[1] Bejerano G, Pheasant M, Makunin I, Stephen S, Kent WJ, Mattick JS, Haussler D (2004). Ultraconserved elements in the human genome. Science.

[2] Cooper GM, Stone EA, Asimenos G, Green ED, Batzoglou S, Sidow A (2005). Distribution and intensity of constraint in mammalian genomic sequence. Genome Res.

[3] Margulies EH, Blanchette M, Haussler D, Green ED (2003). Identification and characterization of multi-species conserved sequences. Genome Res.

[4] Consortium EP (2012). An integrated encyclopedia of dna elements in the human genome. Nature.

[5] Pellegrini M, Marcotte EM, Thompson MJ, Eisenberg D, Yeates TO (1999). Assigning protein functions by comparative genome analysis: protein phylogenetic profiles. Proc Natl Acad Sci.

[6] de Juan D, Pazos F, Valencia A (2013). Emerging methods in protein co-evolution. Nat Rev Genet.

[7] Ochoa D, Pazos F (2014). Practical aspects of protein co-evolution. Frontiers Cell Dev Biol.

[8] Bowers PM, Pellegrini M, Thompson MJ, Fierro J, Yeates TO, Eisenberg D (2004). Prolinks: a database of protein functional linkages derived from coevolution. Genome Biol.

[9] Tillier ER, Charlebois RL (2009). The human protein coevolution network. Genome Res.

[10] Woolfe A, Goodson M, Goode DK, Snell P, McEwen GK, Vavouri T, Smith SF, North P, Callaway H, Kelly K (2004). Highly conserved non-coding sequences are associated with vertebrate development. PLoS Biol.

[11] Fryxell KJ (1996). The coevolution of gene family trees. Trends Genet.

[12] Camacho C, Coulouris G, Avagyan V, Ma N, Papadopoulos J, Bealer K, Madden TL (2009). Blast+: architecture and applications. BMC Bioinformatics.

[13] Jothi R, Cherukuri PF, Tasneem A, Przytycka TM (2006). Co-evolutionary analysis of domains in interacting proteins reveals insights into domain–domain interactions mediating protein–protein interactions. J Mol Biol.

[14] Kim Y, Koyutürk M, Topkara U, Grama A, Subramaniam S (2006). Inferring functional information from domain co-evolution. Bioinformatics.

[15] Yeang C-H, Haussler D (2007). Detecting coevolution in and among protein domains. PLoS Comput Biol.

[16] Pazos F, Valencia A (2001). Similarity of phylogenetic trees as indicator of protein–protein interaction. Protein Eng.

[17] Date SV, Marcotte EM (2003). Discovery of uncharacterized cellular systems by genome-wide analysis of functional linkages. Nat Biotechnol.

[18] Korber B, Farber RM, Wolpert DH, Lapedes AS (1993). Covariation of mutations in the v3 loop of human immunodeficiency virus type 1 envelope protein: an information theoretic analysis. Proc Natl Acad Sci.

[19] Martin L, Gloor GB, Dunn S, Wahl LM (2005). Using information theory to search for co-evolving residues in proteins. Bioinformatics.

[20] Pazos F, Ranea JA, Juan D, Sternberg MJ (2005). Assessing protein co-evolution in the context of the tree of life assists in the prediction of the interactome. J Mol Biol.

[21] Rodionov A, Bezginov A, Rose J, Tillier ER (2011). A new fast algorithm for detecting protein coevolution using maximum compatible cliques. Algoritm Mol Biol.

[22] Yang S, Yalamanchili HK, Li X, Yao K-M, Sham PC, Zhang MQ, Wang J (2011). Correlated evolution of transcription factors and their binding sites. Bioinformatics.

[23] Barbash S, Shifman S, Soreq H (2014). Global coevolution of human micrornas and their target genes. Mol Biol Evol.

[24] Marks DS, Hopf TA, Sander C (2012). Protein structure prediction from sequence variation. Nat Biotechnol.

[25] Hopf TA, Schärfe CP, Rodrigues JP, Green AG, Kohlbacher O, Sander C, Bonvin AM, Marks DS (2014). Sequence co-evolution gives 3d contacts and structures of protein complexes. Elife.

[26] Skwark MJ, Croucher NJ, Puranen S, Chewapreecha C, Pesonen M, Xu YY, Turner P, Harris SR, Beres SB, Musser JM (2017). Interacting networks of resistance, virulence and core machinery genes identified by genome-wide epistasis analysis. PLoS Genet.

[27] Schubert B, Maddamsetti R, Nyman J, Farhat MR, Marks DS (2019). Genome-wide discovery of epistatic loci affecting antibiotic resistance in neisseria gonorrhoeae using evolutionary couplings. Nat Microbiol.

[28] Škunca N, Dessimoz C (2015). Phylogenetic profiling: how much input data is enough?. PloS ONE.

[29] Herman D, Ochoa D, Juan D, Lopez D, Valencia A, Pazos F (2011). Selection of organisms for the co-evolution-based study of protein interactions. BMC Bioinformatics.

[30] Consortium G (2015). The genotype-tissue expression (gtex) pilot analysis: Multitissue gene regulation in humans. Science.

[31] Shabalin AA (2012). Matrix eqtl: ultra fast eqtl analysis via large matrix operations. Bioinformatics.

[32] Ongen H, Buil A, Brown AA, Dermitzakis ET, Delaneau O (2015). Fast and efficient qtl mapper for thousands of molecular phenotypes. Bioinformatics.

[33] Database of Single Nucleotide Polymorphisms (dbSNP). Bethesda (MD): National Center for Biotechnology Information, National Library of Medicine. (dbSNP Build ID: 146). Available from: http://www.ncbi.nlm.nih.gov/SNP/. Accessed: 12 May 2016.

[34] Pollard KS, Hubisz MJ, Rosenbloom KR, Siepel A (2010). Detection of nonneutral substitution rates on mammalian phylogenies. Genome Res.

[35] Rosenbloom KR, Armstrong J, Barber GP, Casper J, Clawson H, Diekhans M, Dreszer TR, Fujita PA, Guruvadoo L, Haeussler M (2015). The ucsc genome browser database: 2015 update. Nucleic Acids Res.

[36] Flicek P, Amode MR, Barrell D, Beal K, Billis K, Brent S, Carvalho-Silva D, Clapham P, Coates G, Fitzgerald S (2013). Ensembl 2014. Nucleic Acids Res.

[37] Kirsten H, Al-Hasani H, Holdt L, Gross A, Beutner F, Krohn K, Horn K, Ahnert P, Burkhardt R, Reiche K (2015). Dissecting the genetics of the human transcriptome identifies novel trait-related trans-eqtls and corroborates the regulatory relevance of non-protein coding loci. Hum Mol Genet.

[38] Siepel A, Bejerano G, Pedersen JS, Hinrichs AS, Hou M, Rosenbloom K, Clawson H, Spieth J, Hillier LW, Richards S (2005). Evolutionarily conserved elements in vertebrate, insect, worm, and yeast genomes. Genome Res.

[39] Davydov EV, Goode DL, Sirota M, Cooper GM, Sidow A, Batzoglou S (2010). Identifying a high fraction of the human genome to be under selective constraint using gerp++. PLoS Comput Biol.

