# Supplementary material for: Characterizing human genomic coevolution in locus-gene regulatory interactions
Source: BioData Min. 2019 Mar 15;12:8. doi: 10.1186/s13040-019-0195-y (PMC6419833; doi:10.1186/s13040-019-0195-y)
Supplement: Supplementary file 1 — Figure S1: First Singular Vector of SVD analysis compared to the High Conservation Centroid of the clustering analysis. Figure S2: Centroids when using different values of K during clustering. Figure S3: Within-Cluster Distance for different values of K during clustering. Figure S4: Distributions of the distance between pairs of SNPs and target gene promoters. (PDF 120 kb) [file 13040_2019_195_MOESM1_ESM.pdf]

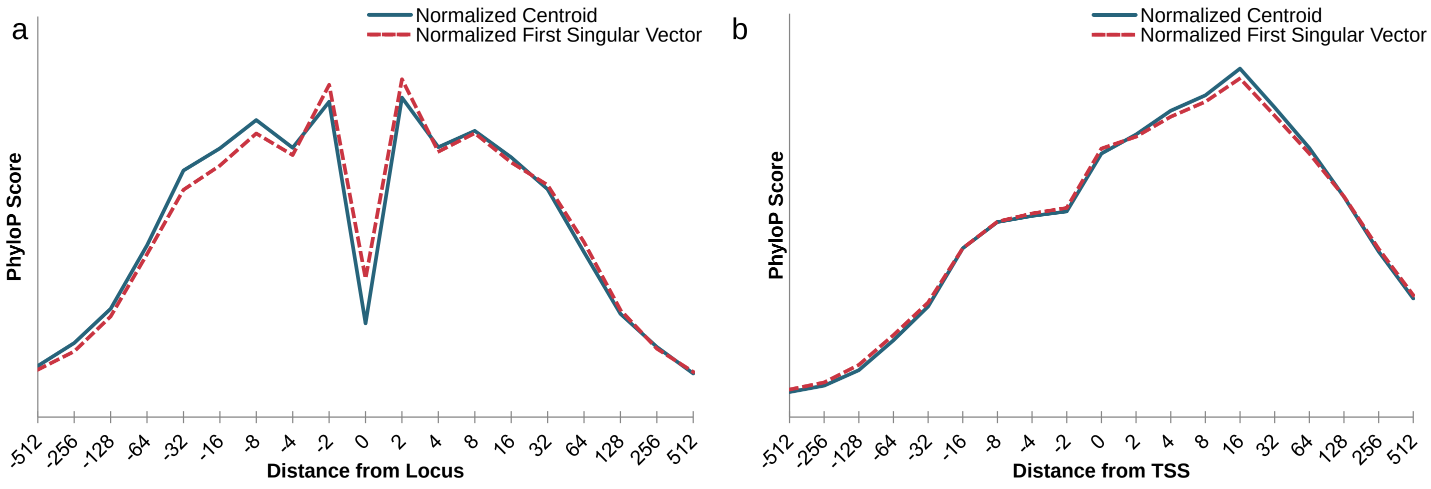

Figure S1: First Singular Vector of SVD analysis compared to the High Conservation Centroid of the clustering analysis

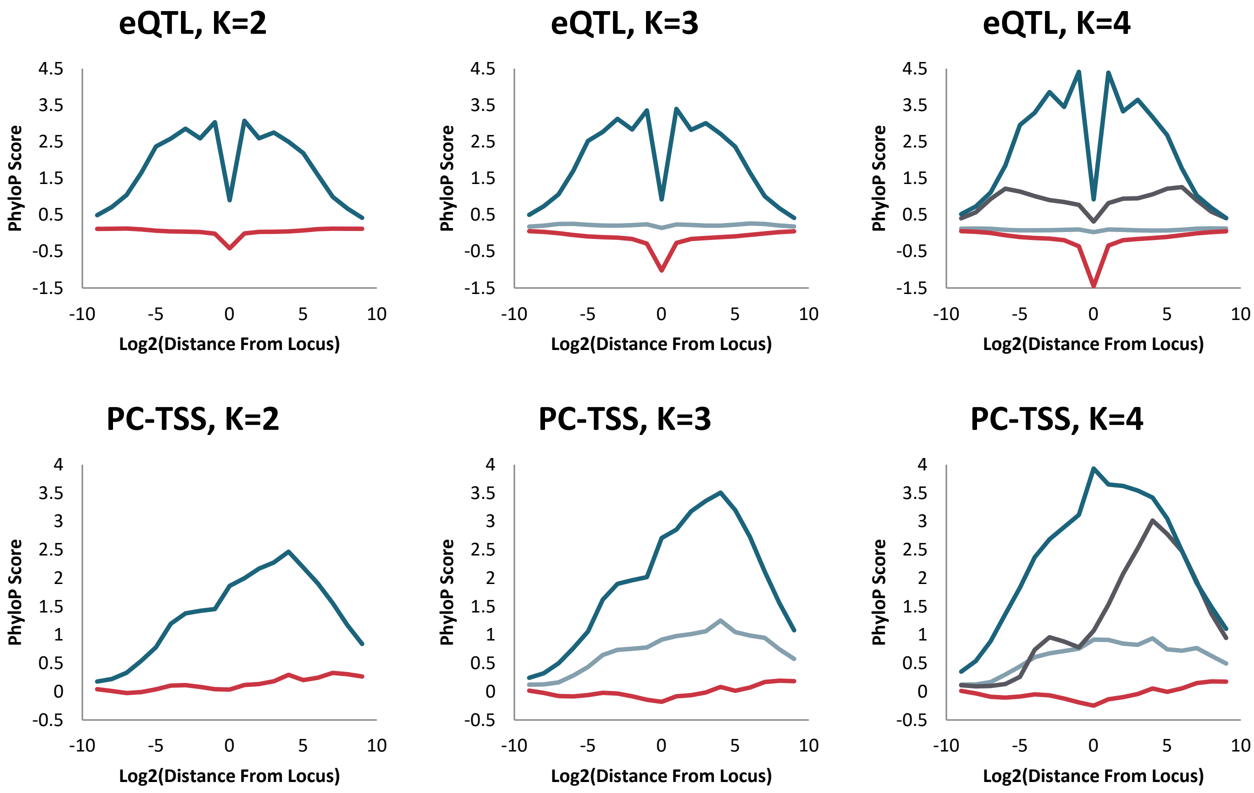

Figure S2: Centroids when using different values of K during clustering

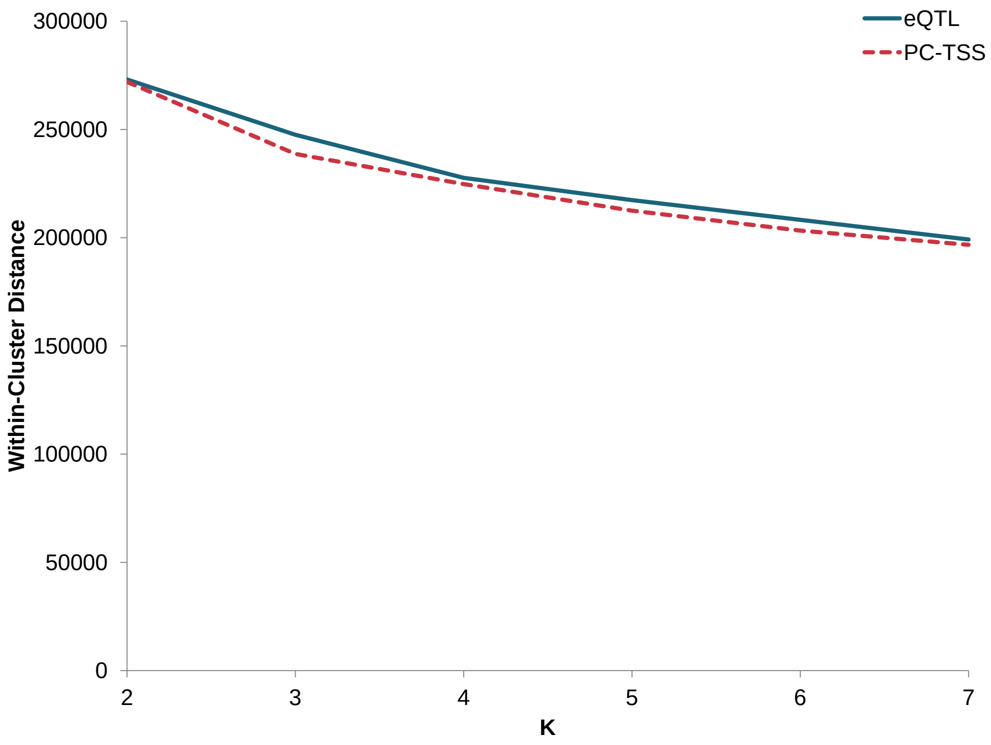

Figure S3: Within-Cluster Distance for different values of K during clustering

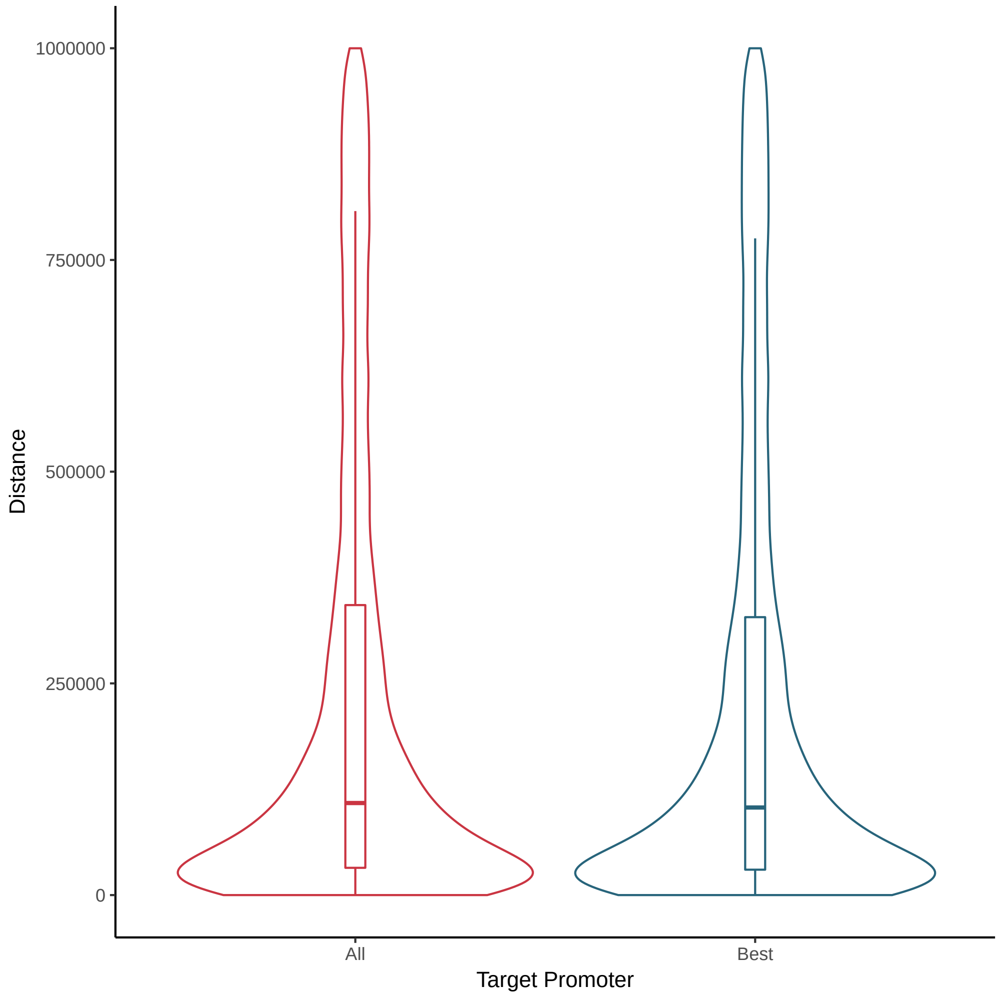

Figure S4: Distributions of the distance between pairs of SNPs and target gene promoters
